# Persistence of Phi6, a SARS-CoV-2 surrogate, in simulated indoor environments: Effects of humidity and material properties

**DOI:** 10.1371/journal.pone.0313604

**Published:** 2025-01-06

**Authors:** Eloise Parry-Nweye, Zhenlei Liu, Yousr Dhaouadi, Xin Guo, Wenfeng Huang, Jianshun Zhang, Dacheng Ren

**Affiliations:** 1 Department of Biomedical and Chemical Engineering, Syracuse University, Syracuse, New York, United States of America; 2 Department of Mechanical and Aerospace Engineering, Syracuse University, Syracuse, New York, United States of America; 3 Center of Excellence in Environmental and Energy Systems, Syracuse, New York, United States of America; 4 Department of Civil and Environmental Engineering, Syracuse University, Syracuse, New York, United States of America; 5 Department of Biology, Syracuse University, Syracuse, New York, United States of America; 6 BioInspired Institute, Syracuse University, Syracuse, New York, United States of America; SKUMS: Shahrekord University of Medical Science, ISLAMIC REPUBLIC OF IRAN

## Abstract

The SARS-CoV-2 virus caused the COVID-19 pandemic and brought major challenges to public health. It is transmitted via aerosols, droplets, and fomites. Among these, viral transmission through fomites is not well understood although it remains a very important transmission route. This motivated us to study how fomites play a role in viral transmission within controlled indoor environments. To achieve this, we investigated viral aerosol persistence on fomites under different humidity levels to mimic the built environment. We developed a protocol to study the effect of humidity on viral infectivity using a full-scale environmental chamber. The results show that the infectivity of aerosolized Phi6 in air decreased by ≥ 1 log_10_ as the relative humidity (RH) increased from 25% to 75% but then increased by ≥ 1 log_10_ as the RH further increased to 85%, resulting in a characteristic V-shape curve which varied with exposure time. Consistently, we show that although material properties may impact viral persistence, changes in the local humidity more significantly influence viral persistence on fomites. These results provide new insights into indoor fomite-mediated viral transmission under different environmental conditions. These findings will help guide the design of more effective strategies for viral control in indoor environments.

## Introduction

The COVID-19 pandemic, caused by the SARS-CoV-2 virus, brought major challenges to the global healthcare system with over 776 million people infected to date. This led to ~7 million deaths globally as of August 18^th^, 2024, including 1.2 million deaths in the U.S. alone [1–3]. Overall, viruses cause approximately 60% of human infections worldwide [4, 5]. The mechanism of airborne viral transmission occurs via the emission and diffusion of virus-laden aerosols (< 5 *μ*m; 5–100 *μ*m) and droplets (> 100 *μ*m) from infected individuals during speaking, coughing, and respiration to susceptible hosts, whereby they cause subsequent infection. In addition, viral transmission is also enabled by direct and indirect contact with different surfaces, formally called fomites [6–14].

By serving as reservoir vehicles, fomites play a critical role in viral infections in indoor environments including offices, homes, classrooms, and hospitals [5, 15–18]. However, viral transmission through fomites, including its persistence on absorptive materials, in indoor environments is not fully understood. The risk of infection from fomites is as significant as that from aerosols and droplets based on a model that utilized data from the SARS-CoV-2 outbreak on the *Diamond Princess* cruise ship. The results showed that each of these transmission routes (i.e., fomites, aerosols and droplets) presents in 30%, 35%, and 35% infection risk respectively [19]. According to Pancic et al. [20], fomite-mediated viral transmission resulted in the recovery of up to 1,800 Plaque-Forming Units (PFU) of infectious rhinovirus from contaminated doorknobs or faucets. Since only 9 PFUs are needed to cause human coronavirus infection (Minimum Infectious Dose, MID) [21, 22], this viral load on contaminated fomites is approximately 200 times of the MID, indicating a significant risk of coronavirus infection from associated surfaces. This motivated the present study with an aim to better understand the role of fomites in indoor viral transmission.

The persistence of viruses on a surface is primarily dependent on the material properties, environmental factors (temperature, humidity, etc.), and characteristics of the viral envelope [23]. Many studies have shown that viruses persist longer on nonporous compared to porous surfaces, also known as nonabsorptive and absorptive surfaces respectively [4, 5, 7, 24–29]. Examples of nonporous surfaces include stainless steel, plastic, glass, aluminum, etc., while porous surfaces include cloth, paper, wood, laboratory coats, facial tissue, hospital gowns, etc. Investigating viral persistence on surfaces has been predominantly conducted using the conventional droplet method, in which viral droplets of 1–10 *μ*L are placed on the test materials and subsequently recovered for analysis. However, there are limitations in utilizing the conventional droplet method to assess viral persistence on surfaces, as it does not reflect realistic conditions [30, 31]. During viral transmission within the built environment, the virus-laden aerosols or droplets are pre-exposed to the surrounding environmental condition of the fomites prior to deposition. Therefore, to gain insight into viral persistence on fomites in real indoor environments, it is important to pre-expose virus-laden aerosols to respective environmental conditions prior to their deposition on test surfaces.

The present study utilized *Pseudomonas* phage Phi6, an 85 nm enveloped double-stranded RNA (dsRNA) virus [32–36], as a coronavirus surrogate due to its biomimetic morphological features. Phi6 has a phospholipid bilayer envelope with spike glycoproteins, which are similar to those of enveloped viruses (SARS-CoV-2, Influenza virus, Ebola virus, etc.) [25, 32, 37–42]. Phi6 is also a BSL-1 agent, and thus safe to work with [41]. It is a bacteriophage that infects the Gram-negative bacterium *Pseudomonas syringae*. Among the 50 pathovars (pv.) of *P*. *syringae*, this study utilized *P*. *syringae* pv. tomato DC3000, which infects tomato plants, its natural host [43].

The environmental factor of primary interest in this study is humidity. The humidity of indoor environments has been shown to affect the stability of viruses [23, 31, 44–47]. Specifically, the infectivity of enveloped viruses including SARS-CoV-2 and Phi6 has been shown to be higher at 20% RH compared to 80% RH [48, 49]. In addition, Phi6 in air has been shown to survive best at < 60% RH and > 85% RH compared to intermediate RH’s of ~60–85% [40, 50]. At RH’s < 60%, Phi6 in air has been shown to survive better at 25% RH compared to 50% RH [51]. However, the effect of humidity on the persistence of deposited viral aerosols on fomites is not well understood.

To gain insight into fomite-mediated viral transmission in indoor environments, it is important to accurately simulate the generation of aerosols under relevant conditions and study how the viruses travel with the aerosols and deposit on fomite materials. It is also important to understand viral persistence on those materials and the impact of humidity and the time of exposure to certain humidity. In this study, we investigated the effect of humidity on the persistence of viral aerosols deposited on fomites using a full-scale chamber (29 m^3^) with well-controlled humidity. We also investigated the effect of humidity on the airborne viral aerosols alongside the deposited viral aerosols to understand viral persistence on surfaces in the built environment. The results provide new insights into viral persistence on surfaces and the infection risk from fomite-mediated viral transmission in the built environment. This method can also be used to evaluate antiviral materials.

## Materials and methods

### Phi6 propagation and plaque assays

Bacteriophage Phi6 was obtained from Laboratoire de Sylvain Moineau, Université Laval, Québec, Canada. To propagate the phage, 0.5 mL of a 16-h *P*. *syringae* pv. tomato DC3000 culture and 0.5 mL of reconstituted Phi6 were mixed into 50 mL of sterile 10 mM MgCl_2_ –Tryptic Soy Broth (TSB; Becton Dickinson 211825). The mixture was then incubated with aeration at 25°C and 200 rpm agitation overnight. Propagated Phi6 was harvested by centrifugation at 10,500 rpm for 5 mins to pellet host cells and debris, followed by filtration through a 0.22 *μ*m filter. The filtered virus stocks were stored at 4°C and had a concentration of 10^11^ PFU/mL.

Plaque assays were used to quantify the infectious viral titers. Briefly, a serial 10-fold dilution of the viral sample was prepared in phage buffer [50 mM Tris-HCl pH 7.5 + 100 mM NaCl + 8 mM MgSO_4_]. Next, 3 mL of prewarmed (50°C) 0.6% soft agar TSB solution was inoculated with 0.1 mL *P*. *syringae* pv. tomato DC3000 and the serially diluted viral sample. The suspension was quickly mixed and poured onto TSB agar plates using the double agar overlay method [52]. The plates were incubated at 25°C for 16–24 h, i.e., till the plaques became visible. The PFU was counted, multiplied by the dilution coefficient, and normalized by the volume of diluted phage to determine the infectious viral titer as PFU per milliliter.

### RNA extraction and cDNA synthesis

To quantify the total viral load, RNA was extracted from 0.14 mL of each sample using the QIAamp Viral RNA Mini Kit (Qiagen LLC, MD) according to the manufacturer’s protocol. Next, the isolated RNA was reverse transcribed to cDNA using the iScript cDNA synthesis kit (Bio-Rad Laboratories, Hercules, CA). The cDNA synthesis reaction mix comprised of 5 *μ*L of Nuclease-free water, 4 *μ*L of 5x iScript Reaction Mix, 10 *μ*L of extracted RNA, and 1 *μ*L of iScript Reverse Transcriptase. The cDNA synthesis reaction was performed according to the manufacturer’s protocol using a thermal cycler (C1000 Touch Thermal Cycler, Bio-Rad Laboratories, Hercules, CA).

### qPCR analysis

The copy number of the target gene (*phi-6S_1*) from the synthesized cDNA was quantified using the SYBR Green dye-based qPCR method. The qPCR reaction mix comprised of 4 *μ*L Nuclease-free water, 5 *μ*L cDNA template, 1 *μ*L forward and reverse primer mix (Table 1), and 10 *μ*L SYBR Green dye. The qPCR reaction was performed using a thermal cycler (Quantstudio^™^ 3 Real-Time PCR System, Applied Biosystems^™^). Lastly, absolute quantification of the Viral Genome Copies per milliliter was determined by interpolation, using the threshold cycle (C_T_) values from targeted amplification and standard curve (S3 Fig).

**Table 1:** Primers used for real-time qPCR.

Phage	Primer type	Sequence^a^	Reference
**ϕ6**	**Forward**	5’-TGGCGGCGGTCAAGAGC-3’	[32]
	**Reverse**	5’-GGATGATTCTCCAGAAGCTGCTG-3’	

^a^ to target the *phi-6S_1* gene (coding for the outer capsid P8 protein) located on the S segment

### Small-scale aerobiology chamber

A 50 L small-scale aerobiology chamber was used to optimize the sampling method for aerosolized Phi6. Using a syringe pump, Phi6 was injected into an 8-jet BLAM nebulizer (CH Technologies Inc.) at an injection rate of 0.3 mL/min. The liquid feed was then aerosolized into the small-scale chamber using the influx of compressed air at a rate of 23 L/min. An Aerodynamic Particle Sizer (APS) was used to measure the particle size distribution and concentration during aerosolization. The operating conditions including relative humidity and temperature were recorded. Upon attaining steady state (S4 Fig), the viral aerosols were sampled into 20 mL TSB using the BioSampler (SKC, Inc; 225–9595). The air samples were stored at 4°C and analyzed using plaque assays and qPCR. The viral concentrations in air were calculated using Eq 1 below according to Turgeon et al. [38]:

$$V_{air}=\frac{C_{air}\;\times \;v}{f\;\times \;t}$$ (1)

In Eq 1, *V*_*air*_ is the airborne concentration of the aerosolized virus sampled by the BioSampler (i.e., PFU or viral genome copies/L of air); *C*_*air*_ is the viral concentration in the air sample (PFU or viral genome copies/mL); *v* is the liquid volume in the air sampler (mL); *f* is the air sampling flow rate (Liters/min); *t* is the sampling period (min).

### Full-scale aerobiology chamber

A 29 m^3^ full-scale aerobiology chamber was used to investigate the effect of humidity and exposure time on aerosolized Phi6. The chamber was designed to mimic a realistic building thermal environment. However, to eliminate the effect of ventilation, the chamber was airtight. Four relative humidity conditions including 25%, 45%, 75%, and 85% RH as well as exposure times of 0 min, 30 min, 60 min, 90 min, and 120 min were tested. The viral injection point was located near the mouth of a mannequin, and air samples were collected 1 ft away from the injection point (S1 Fig) as the sampling point. Additionally, the effect of humidity on deposited Phi6 was evaluated using 1.5 cm by 1.5 cm coupons of building materials in triplicates. The coupons were adjacently distributed on a collection tray placed between the injection point and sampling point. The chamber and materials were equilibrated overnight at the tested humidity condition prior to the start of Phi6 aerosolization.

To achieve a high initial concentration of aerosolized viruses, 55 mL of a 10^11^ PFU/mL Phi6 stock was continuously injected for 3 h with two mixing fans at opposite corners of the chamber to ensure a well-mixed condition. Immediately after the viral injection stopped, the first air sample was collected, and taken as the 0 min reference. Subsequently, four additional air samples were collected at 30 min, 60 min, 90 min, and 120 min, respectively. The viral aerosols were sampled into 20 mL TSB using the SKC BioSampler at 11.5 LPM for 15 min sampling periods. After the 120 min air sample was collected, a hazmat suit was worn to recover the deposited viruses from the materials into 1 mL TSB. All samples were stored at 4°C before being analyzed using plaque assays and qPCR.

### CHAMPS-BES simulation

This study employed the CHAMPS-BES (Coupled Heat, Air, Moisture and Pollutant Simulation in Building Envelope Systems) model [53] to simulate the experimental conditions. Specifically, this simulation represented the introduction of 5 *μ*L water droplets onto the top surface of a 15 mm × 15 mm × 10 mm porous material. The simulation domain was the volume of the material, with the top surface applied as a vapor diffusion interface. The exchange coefficient for vapor diffusion was determined based on the air flow rate within the chamber. This coefficient has been previously measured under the same settings, as reported by Zhang et al. [54]. The other surfaces except the top, were defined as adiabatic and impermeable. The air temperature and RH of the chamber were set as 23°C and 45%, respectively. The initial condition is the same as the air condition due to overnight conditioning of the material during the test.

### Statistical analysis

Significant differences in infectivity and viral load between experimental conditions were determined using one-way ANOVA followed by the Tukey test. Data with a *p*-value of < 0.05 are considered as statistically significant.

## Results

### Optimizing the sampling protocol for chamber tests

In this study, we used a full-scale chamber that has an appropriate setup to mimic the real indoor environment and a size that is sufficient to allow effective air circulation to mimic the real environment as well. Specifically, we simulated the built environment by using a 29 m^3^ full-scale environmental chamber, which is ~1,000× larger than the traditional 27 L Goldberg chamber [40, 50, 55]. Additionally, the full-scale chamber has the capability to host built-in injection and sampling systems for the virus-laden aerosols to be delivered, well-mixed with the controlled humidity during the time of exposure, and then sampled at desired points in the chamber. Also, the chamber is large enough to allow the fomite samples to be placed on different surface locations to mimic the indoor environment. This enables us to systematically investigate the impact of humidity and exposure time on aerosol-carried Phi6 as a surrogate to understand SARS-CoV-2 transmission in the indoor environment and the role of fomites.

**Fig 1:**
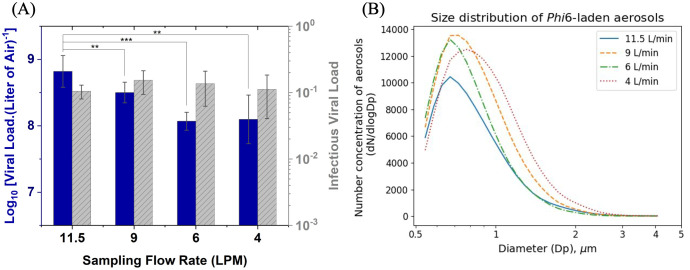
Higher sampling flow rates result in higher viral recovery. (A) Concentration of viruses recovered from air (including total viral load and infectious viral load) with different sampling flow rates (n = 3). Lower flow rates (6 L/min & 4 L/min) resulted in less viral particles recovered compared to higher flow rates (11.5 L/min & 9 L/min). Infectious viral load across the sampling flow rates did not show significant variation (*p* > 0.05). (B) Peak particle sizes in the particle size distribution were < 5 *μ*m, which confirms the presence of Phi6-laden aerosols.

We first optimized the air sampling flow rate for the test protocol. High flow rates can increase sampling efficiency but may also have a negative impact on the integrity and infectivity of viruses. Thus, we first varied the air sampling flow rate to assess the effect on aerosolized Phi6 recovery and infectivity. We aimed to identify an optimal air sampling flow rate that recovers relatively high viral loads without compromising the infectivity of the virus. The tested air sampling flow rates include 11.5 L/min (close to the capacity limit), 9 L/min, 6 L/min, and 4 L/min, and the experiment was conducted in a small-scale 50 L environmental chamber. The higher air sampling flow rate of 11.5 L/min resulted in a 1 log_10_ higher viral load recovered compared to the lower air sampling flow rates of 6 L/min and 4 L/min respectively (Fig 1A). The infectious viral load did not vary significantly (*p* > 0.05). Based on these results, 11.5 L/min air sampling flow rate was selected to sample aerosolized Phi6 in subsequent experiments.

### Effect of humidity and exposure time on aerosolized Phi6 in a 29 m^3^ full-scale aerobiology chamber

**Fig 2:**
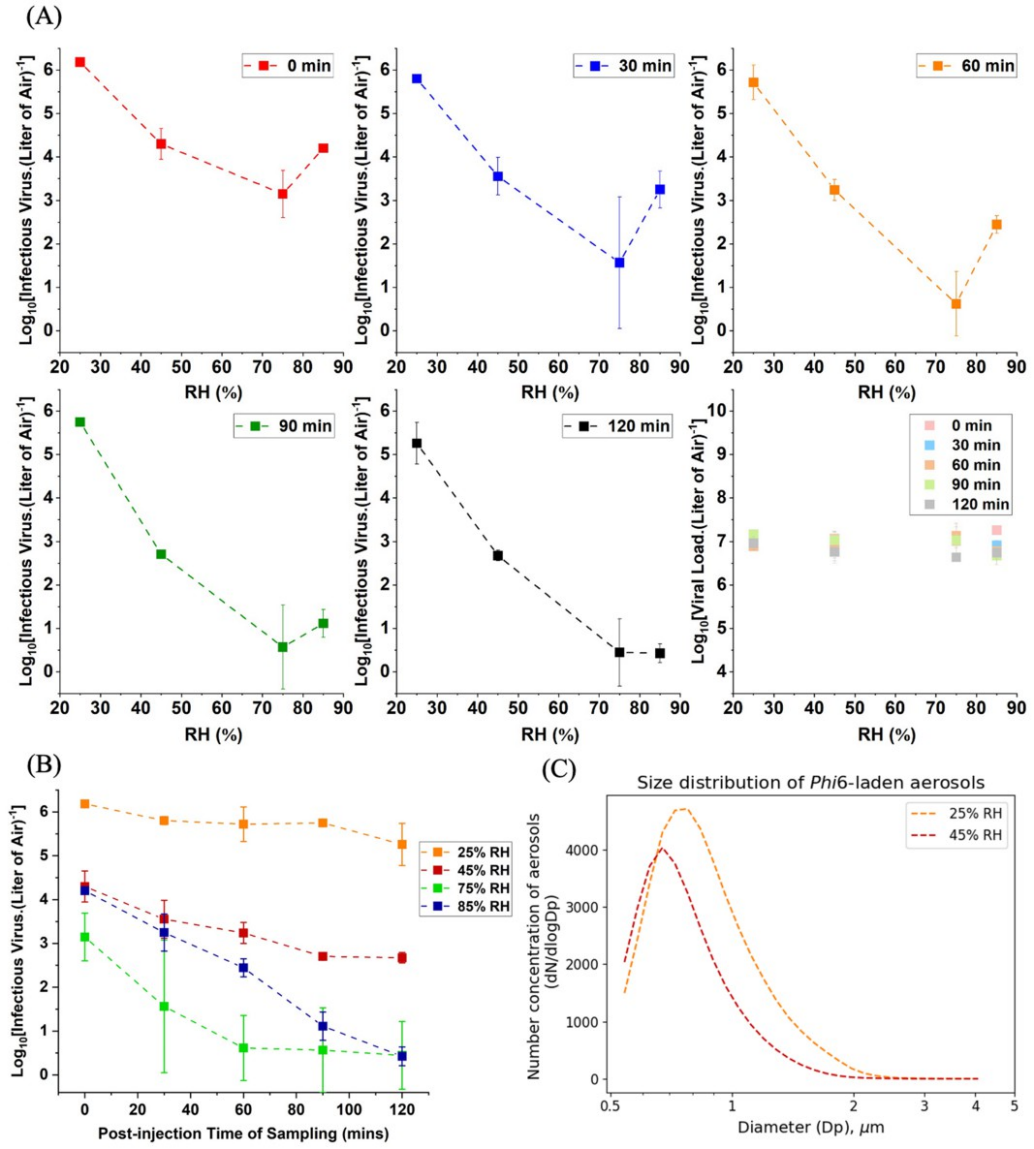
Persistence of aerosolized Phi6 in air. (A) Effect of relative humidity (RH) on the infectivity of aerosolized Phi6 follows a characteristic V-shape which varies over time. (B) The infectious viruses recovered as a function of exposure time under tested humidity conditions. (C) Size distribution of Phi6-laden aerosols. All conditions were tested in duplicate.

We investigated the time-dependent effect of humidity on aerosolized Phi6 in a 29 m^3^ full-scale environmental chamber. The tested conditions include relative humidity of 25%, 45%, 75%, and 85% RH at room temperature (around 25°C). At all five exposure times, the infectivity of Phi6 was consistently highest at 25% RH compared to other RH conditions by ≥ 1 log_10_. At exposure times from 0 h to 1 h, the infectivity decreased and then increased with RH (Fig 2A), representing a characteristic humidity-induced V-shape pattern of aerosolized Phi6 infectivity. However, Phi6 infectivity was found to be most susceptible between 75–85% RH in this study, as the infectivity was completely lost with prolonged exposure times. The results therefore indicate that there is a time-dependency of the humidity-induced V-shape pattern of aerosolized Phi6 infectivity. Furthermore, the viral load, averaged in 7 log_10_ for the air samples, validated our experimental method which included aerosolizing 55 mL from the same 10^11^ PFU/mL stock across all the humidity experiments. In addition, the results show that higher humidity did not affect the viral load in aerosols.

### Viral persistence on materials differs between loaded droplets and deposited aerosols

**Fig 3:**
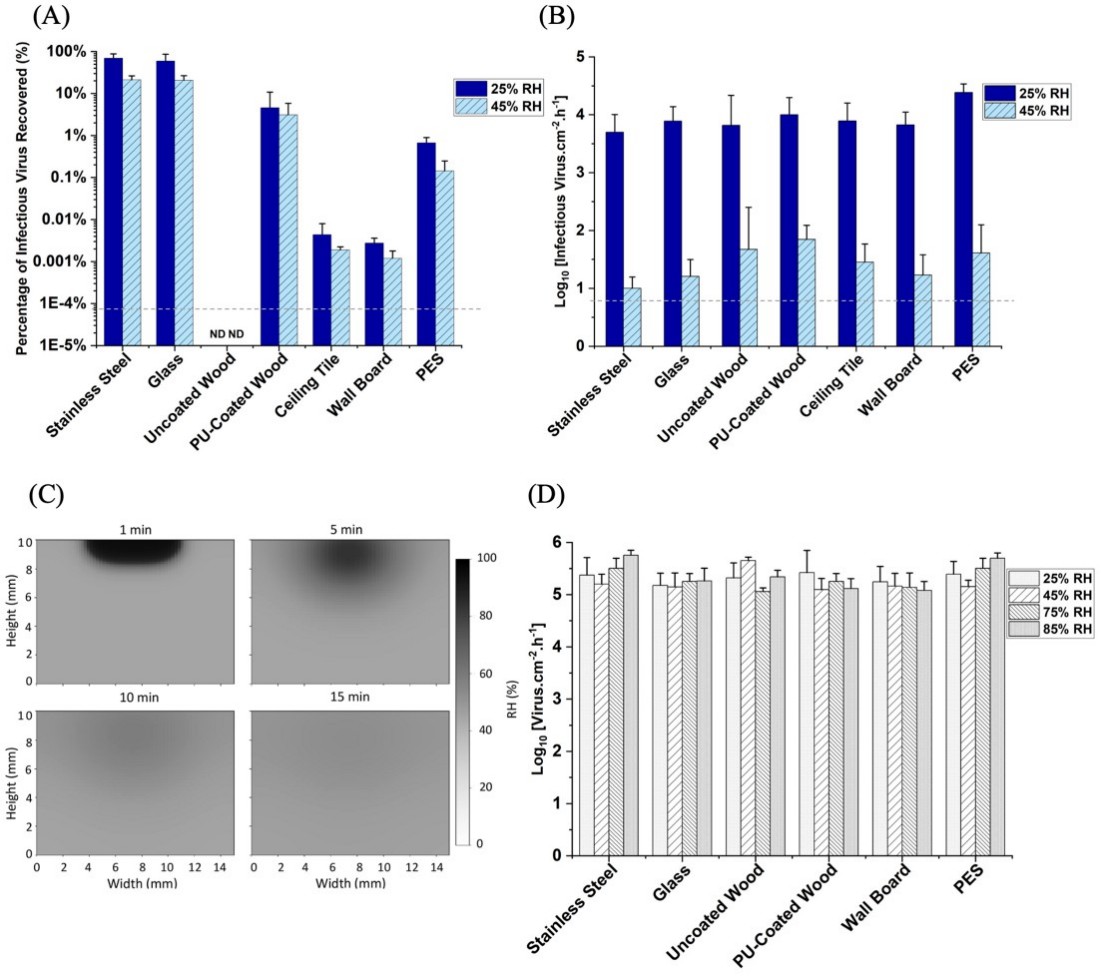
Persistence of Phi6 on materials. (A) Phi6 is pre-loaded on materials as conventional droplets (n = 3). (B) Phi6 is aerosolized followed by deposition on materials (n = 6). Dashed lines indicate the limit of detection. (C) RH changes within an absorptive material (gypsum wallboard as an example). (D) Deposited viral load on materials in the full-scale chamber (n = 3).

To understand viral infectivity on fomites, we evaluated the effects of humidity and material properties on Phi6 infectivity. We inoculated the surfaces of different building materials with Phi6 as droplets or deposited aerosols. For the conventional droplet method, 5 *μ*L aliquots of Phi6 were loaded onto pre-conditioned materials and exposed to the selected humidity for 6 h in a small-scale environmental chamber (S2 Fig). Conversely, we exposed similar pre-conditioned materials to deposited Phi6 aerosols at the same humidity conditions for 6 h in a 29 m^3^ full-scale environmental chamber (S1 Fig). We observed that when the virus was loaded on the materials as droplets, there was a 1–4 log_10_ decrease in Phi6 infectivity on the porous materials compared to the nonporous materials. This is consistent with other reports that porous materials reduce viral infectivity [24–26, 29]. However, when the same set of materials was exposed to Phi6 aerosols, the infectivity between the samples did not vary significantly irrespective of material properties. These results showed that the local humidity of the porous materials significantly varied from the nonporous materials when the materials were exposed to Phi6 droplets (Fig 3C). In contrast, the local humidity of both the porous and nonporous materials was similar when the materials were exposed to Phi6 aerosols as we observed that the local humidity of the materials was at an equilibrium with the surrounding humidity. Specifically, the 2 log_10_ decrease in infectivity between 25% RH and 45% RH of the deposited Phi6 (Fig 3B) is consistent with the 2 log_10_ decrease in infectivity between 25% RH and 45% RH of the aerosolized Phi6 in air (Fig 2B). Furthermore, we observed that the effect of 45% RH on Phi6 as aerosols resulted in ≥ a 2 log_10_ decrease in infectivity (Fig 3B) which is significantly higher compared to the < 1 log_10_ decrease in infectivity when Phi6 was as droplets (Fig 3A). These new findings emphasize the importance of using aerosols to simulate viral transmission in indoor environments.

## Discussion

The persistence (i.e., time-dependent infectivity) of viruses in the built environment determines the risk of viral infection. The longer the virus persists within the environment, the greater the risk of infection via the primary routes of viral transmission. Previous studies have shown that the persistence of viruses in indoor environments is significantly influenced by humidity [23, 31, 44–46, 56–60]. However, the effects of humidity on transmission via fomites and the role of fomite properties are not fully understood. In addition, loading viruses in droplets on a surface does not mimic viral transmission in real indoor environments. Fig 2A shows that as the RH increased from 25% to 75%, the infectivity of aerosolized Phi6 decreased by 3 orders of magnitude and 5 orders of magnitude when the exposure times were 0 h and 2 h respectively. These results revealed that humidity is a key determinant of the risk of viral infection in the built environment. To gain further insights into the risk of viral infection from fomites, we investigated the effect of humidity on viral aerosol persistence on fomites. This investigation also provides new knowledge about the infection risk from deposited aerosols of enveloped viruses such as SARS-CoV-2.

### Humidity-dependent persistence of aerosolized Phi6

Previous studies on humidity and infectivity of aerosolized Phi6 were conducted in either a 27 L or 55 L aluminum Goldberg drum [40, 49–51, 55]. In addition, Skanata et al. [61] investigated the effect of humidity on the dispersion of aerosolized Phi6 in a classroom to determine long-range airborne viral transmission in closed spaces. This is a realistic environment; however, it does not provide rigorously regulated conditions for repeated experiments. Thus, it is important to study the effect of humidity on the persistence of aerosolized Phi6 in a full-scale (29 m^3^) environmental chamber with well-controlled aerosol generation. This chamber (S1 Fig) was designed to adequately simulate the built environment by situating a thermal mannequin in a test room with carpet flooring, painted gypsum wallboards, ceiling, office furniture, and a laptop. Aerosol generation was used to best mimic viral dispersion from infected individuals.

Our results show that Phi6 infectivity was consistently higher at 25% RH compared to all other tested humidity conditions across all exposure times (Fig 2A and 2B). Several studies have reported that the infectivity of enveloped viruses, including strains of influenza virus and SARS-CoV-2, is better retained at lower RH compared to intermediate RH [25, 40, 48–51, 62, 63]. Our results from the full-scale chamber are consistent with those of other systems in literature, and demonstrate that the risk of viral infection in indoor environments is greatest at lower humidity levels. When the RH was increased from 25% to 45%, we observed that the infectivity decreased by 2 orders of magnitude and 3 orders of magnitude at 0 h and 0.5–2 h exposure times respectively. Similarly, Noti et al. [64] investigated the effect of humidity on the infectivity of aerosolized influenza virus in a 23 m^3^ chamber and showed that the infectivity decreased by ~0.5 log_10_ when the RH was increased from 20% to 45%. With prolonged exposure time at 45% RH, we observed up to a 1.5 log_10_ decrease in infectivity from 0–1 h followed by a plateau in infectivity from 1.5–2 h. This is consistent with the observation of a plateau in the infectivity of aerosolized SARS-CoV-2 at 40% RH by Oswin et al. [65].

Beyond the lower humidity extremities, we investigated the effect of higher humidity conditions on Phi6 infectivity. We observed that the infectivity at 75% RH was the lowest compared to the infectivity at 25–45% RH and 85% RH for exposure times between 0–1 h. Our results are consistent with earlier reports [40, 50, 55], which showed that aerosolized Phi6 infectivity is consistently the lowest at 75% RH after 1 h of exposure time. In addition, unlike the infectivity at 25–45% RH which persisted with prolonged exposure times, we found that Phi6 was most susceptible to 75–85% RH, as infectivity was completely lost with prolonged exposure times of 1.5–2 h. Similarly, Oswin et al. found that only 10% of aerosolized SARS-CoV-2 remained infectious when exposed to 90% RH for 10 min [65]. These findings can help develop disinfection strategies of humidification to control airborne viral transmission in the built environment. Based on our observations of the humidity-induced viral inactivation, we investigated the kinetics of decay. We found that the humidity-induced inactivation of Phi6 at 25%, 45% and 75% RH follows first-order kinetics (S5 Fig), which is typical for viral decay kinetics [31]. More specifically, the decay rate constant at 75% RH was ~2× greater than other humidity levels, indicating that prolonged exposure of infectious viral aerosols under higher humidity conditions results in rapid viral inactivation.

We observed the characteristic V-shaped curve for exposure times from 0–1 h. However, the V-shape was minimally defined with exposure times of 1.5–2 h due to the complete inactivation of Phi6 at 75–85% RH with prolonged exposure time. Verreault et al. [49] investigated the effect of 20%, 50%, and 80% RH on aerosolized Phi6 infectivity for 0 h, 6 h and 14 h exposure times, at 18°C in an aluminum Goldberg drum [66]. Different from other studies, we focused on the time-dependency of the humidity-induced V-shape pattern of aerosolized Phi6 infectivity at standard ambient temperature in a simulated built environment. We sampled the air at the exposure times of 0, 0.5, 1, 1.5, and 2 h to investigate the persistence of the virus in the air immediately after the release of virus-laden aerosols in poorly ventilated spaces. After 1 h exposure of aerosolized Phi6 to the desired humidity, we observed a similar V-shape with the reported literature [40, 50], in which aerosolized Phi6 was also aged for 1 h at varied RH. The V-shape curve, also observed by Niazi et al. [67], represents a decrease in infectivity followed by an increase in infectivity as humidity increases. However, Oswin et al. [65] did not observe the characteristic V-shape when the effect of humidity was investigated on aerosols of SARS-CoV-2. Regardless, at our exposure time of 0.5 h, the infectivity of aerosolized Phi6 at 45% RH and 85% RH were comparable. Similarly, at an exposure time of 0.33 h, the infectivity of aerosolized SARS-CoV-2 at 40% RH and 90% RH were likewise comparable [65]. This emphasizes the importance of experimental methods including media for viral suspensions [68], viral species studied, exposure time, etc. in assessing the persistence of viruses. Since people spend 90% of their time indoors [69–71], it is promising to mitigate airborne and fomite-mediated viral disease transmission in indoor environments by leveraging optimal RH conditions.

### Viability of Phi6 on surfaces as droplets vs. aerosols

Previous studies that utilized viral droplet inocula reported that coronaviruses persist longer on nonporous surfaces compared to porous surfaces [72–74]. In this study, we show that viral persistence on nonporous and porous surfaces is, however, more significantly impacted by the viral state (i.e., droplets vs. aerosols). As droplets, we found that Phi6 infectivity decreased by 1–4 log_10_ on the porous materials (wood, ceiling tile, gypsum board, and polyethersulfone) compared to the nonporous materials (stainless steel and glass), similar to earlier reports [72–74]. Loss of viral infectivity on porous materials has been attributed to droplet evaporation kinetics, droplet drying time, and permeability of the fomite material [75–77]. Additionally, there have been speculations that the trapping of viral particles within the porous materials results in insufficient elution [26, 75]. By vortexing, we effectively recovered Phi6 from all the materials tested in this study (S6 Fig). Thus, the sharp contrast in results between loaded droplets and deposited aerosols is not due to sampling but rather provides new insights into the role of humidity.

As aerosols, we show that viral infectivity on both nonporous and porous surfaces was similar. Our results highlight the significance of assessing viral stability on materials via viral aerosols given that fomite-mediated viral transmission is dependent on viral aerosol transmission and deposition. We also found that there was a similar effect of humidity on deposited viral aerosols compared to the aerosols in air. Specifically, there was a consistent 2 log_10_ decrease in infectivity between 25% RH and 45% RH on the deposited viral aerosols (Fig 3B) and the aerosols in air (Fig 2B).

Furthermore, we observed that humidity-induced inactivation is limited by viral state (i.e., droplets vs. aerosols). Notably, the effect of 45% RH on Phi6 aerosols resulted in ≥ a 2 log_10_ decrease in infectivity (Fig 3B) which was significantly larger compared to the < 1 log_10_ decrease in infectivity of Phi6 droplets (Fig 3A). According to Bhardwaj et al. [44], higher humidity increases viral survival in droplets more than viral survival in aerosols, which suggests that viral aerosols are more susceptible to humidity-induced inactivation compared to viral droplets.

**Fig 4:**
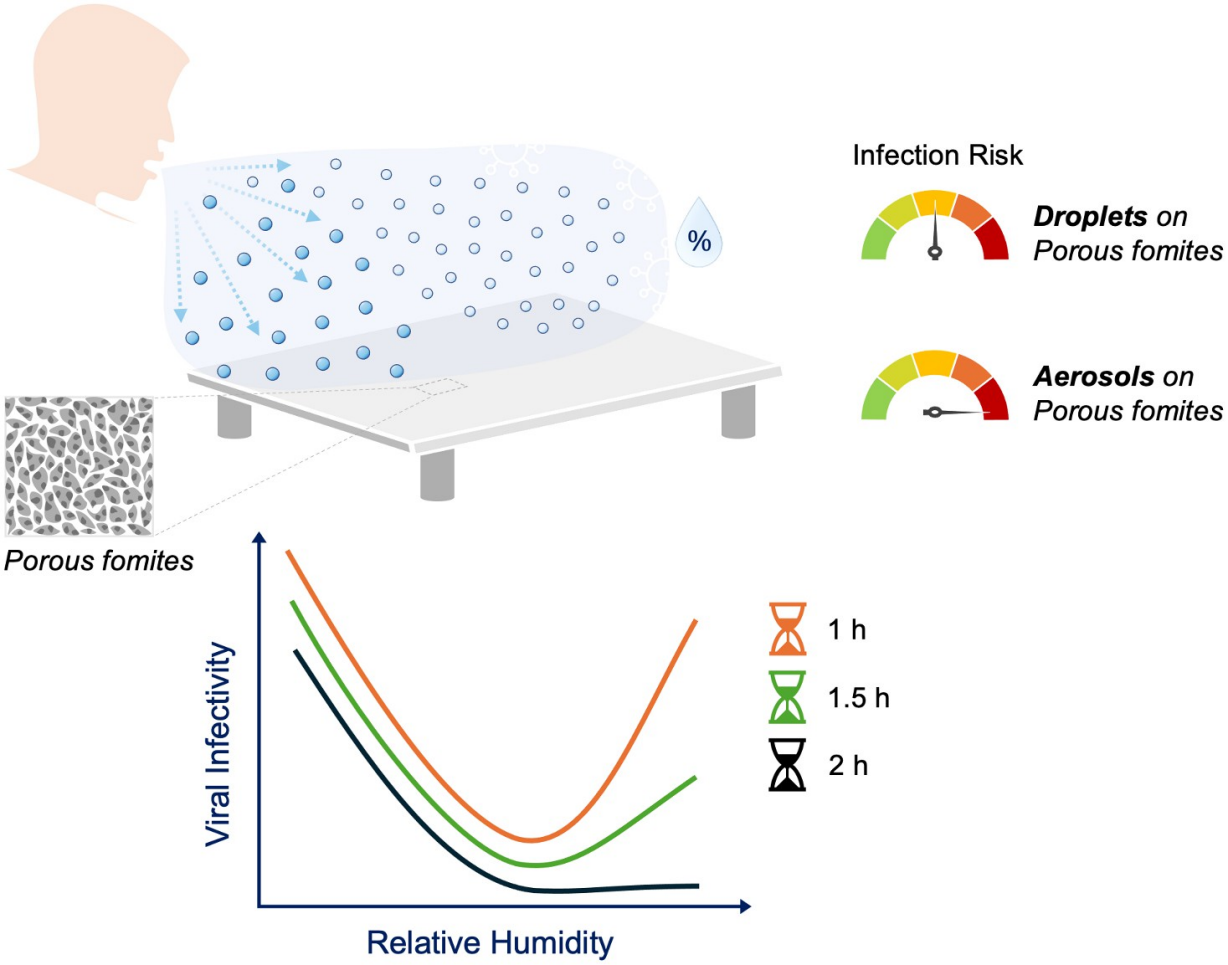
A schematic depicting the risk of infection from deposited virus-laden droplets and aerosols on porous fomites.

Our new results also suggest that the risk of viral infection from porous materials may be underestimated due to viral droplet inoculation. A schematic summarizing these new findings is shown in Fig 4. When aerosols and fomites were pre-exposed to the same humidity of air, the effects of porosity diminished compared to what was observed for viruses in droplets. Thus, a more accurate assessment of viral persistence on materials should be conducted with viral aerosols.

### Needs for future work

This study used Phi6 as a coronavirus surrogate due to its biomimetic characteristics and BSL-1 suitability. However, the genomic construct of Phi6 and SARS-CoV-2 are distinct as Phi6 has a double-stranded RNA genome while SARS-CoV-2 has a single-stranded RNA genome. This difference in addition to genomic variations may affect the persistence of these viruses under different conditions. Further studies are needed to better understand the differences. In addition, the sampling methods and qPCR used in this study are low throughput. New sensors that are able to detect viruses in real time will significantly improve data collection and thus enable more comprehensive analysis.

## Conclusions

The results of this study reveal new information about viral load and infectivity in built environments. Effective sampling of aerosolized Phi6 can be achieved with short sampling periods of 15 mins and high sampling flow rates of 11.5 L/min. The effect of humidity on the infectivity of aerosolized Phi6 in air follows a characteristic V-shape curve, which varies over time; and the humidity-induced inactivation at 75% RH is faster than other tested humidity levels. In addition, we found a similar effect of humidity across the airborne and deposited viral particles. Viral persistence on materials is humidity-dependent. Notably, when local humidity was the same across porous and non-porous materials, viral infectivity was found similar among these samples, indicating that porous materials pose a similar risk of viral infection as non-porous materials. These results provide new insights into infectious disease transmission and emphasize the importance of using controlled aerosol generation to study viral transmission via fomites.

## Supporting information

S1 Fig Pictures of the 29 m^3^ full-scale environmental chamber. (TIFF)

S2 Fig A picture of the 50 L small-scale environmental chamber. (TIFF)

S3 Fig qPCR standard curve from a pure and concentrated (10^9^ PFU/mL) Phi6 stock. (TIFF)

S4 Fig Particle concentration at different air sampling flow rates. (TIFF)

S5 Fig Humidity-induced Phi6 inactivation at 25%, 45% and 75% RH follows first-order kinetics. (TIFF)

S6 Fig Viral recovery efficiency from fomites by vortexing. (TIFF)
