# Supplementary figures and images for: Persistence of Phi6, a SARS-CoV-2 surrogate, in simulated indoor environments: Effects of humidity and material properties
Source: PLoS One. 2025 Jan 6;20(1):e0313604. doi: 10.1371/journal.pone.0313604 (PMC11703003; doi:10.1371/journal.pone.0313604)

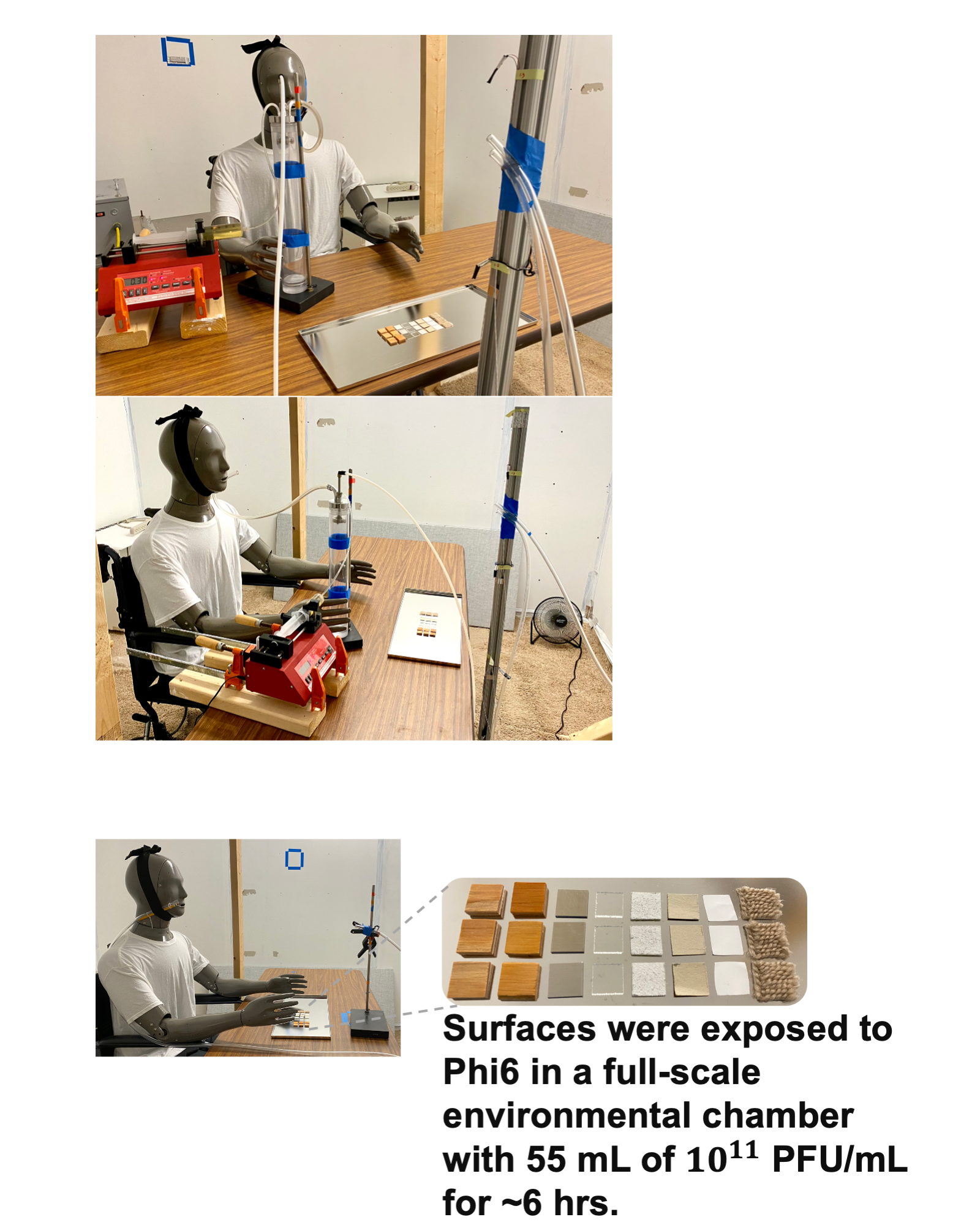

Supplement: S1 Fig — (TIFF) [file pone.0313604.s001.tiff]

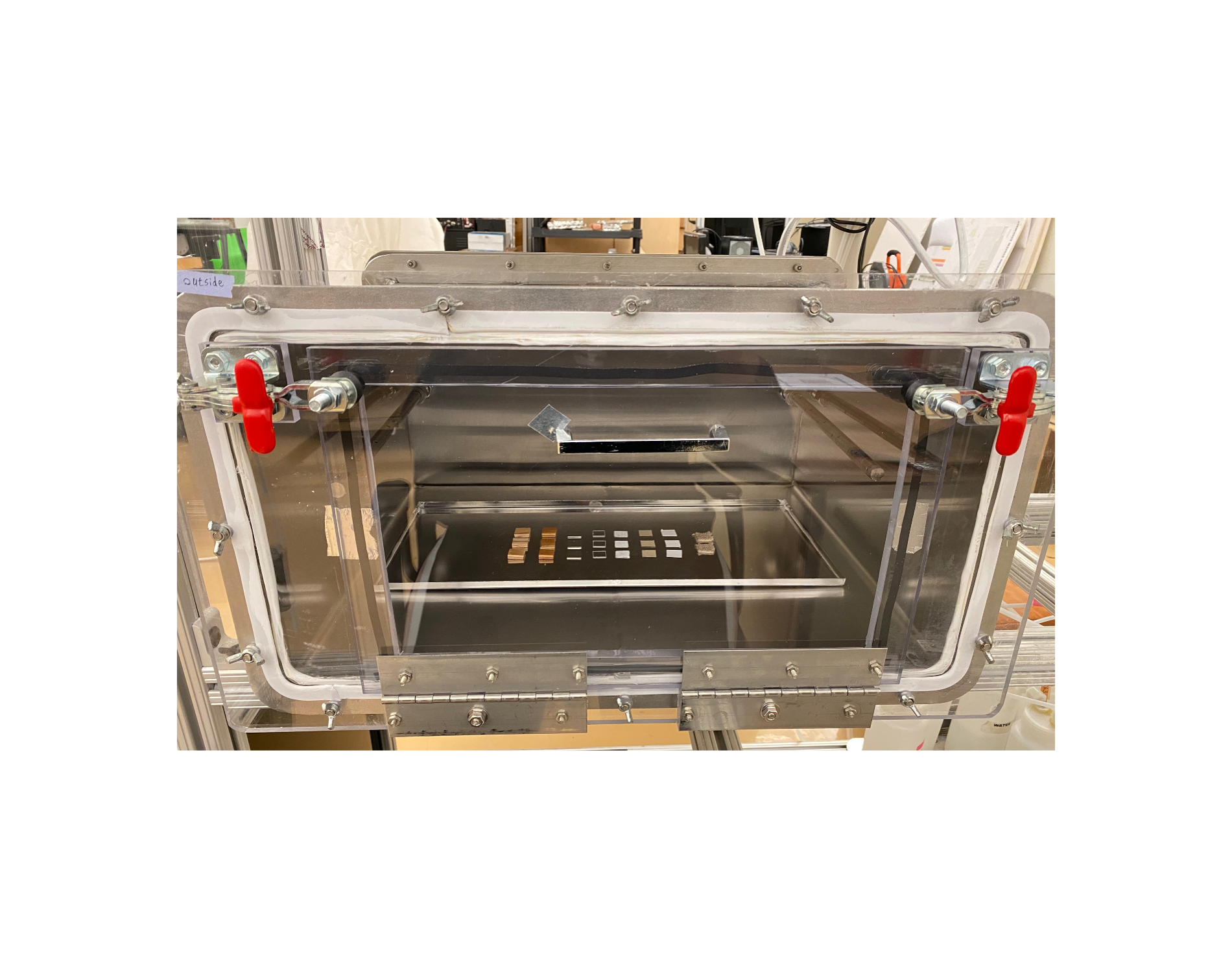

Supplement: S2 Fig — (TIFF) [file pone.0313604.s002.tiff]

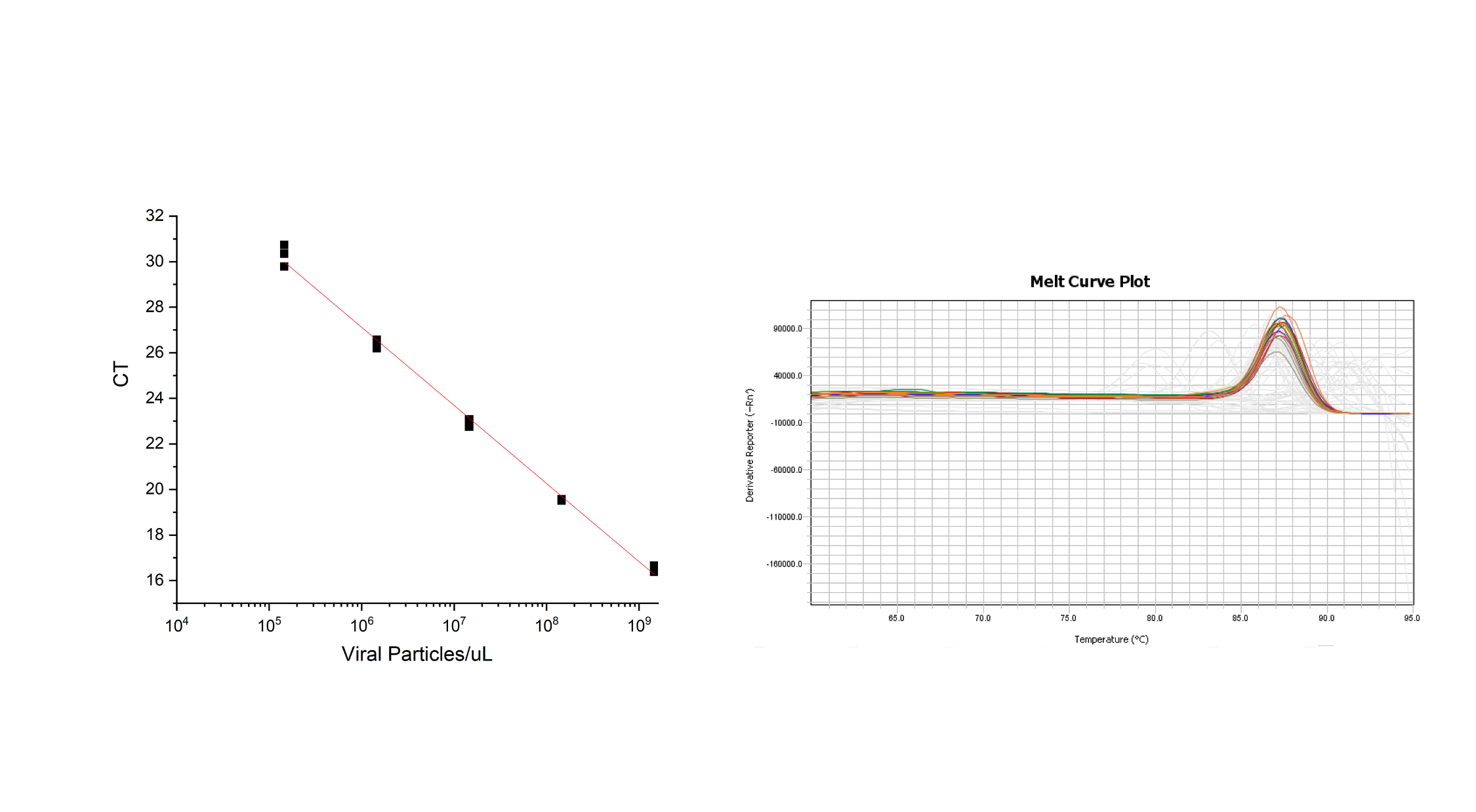

Supplement: S3 Fig — (TIFF) [file pone.0313604.s003.tiff]

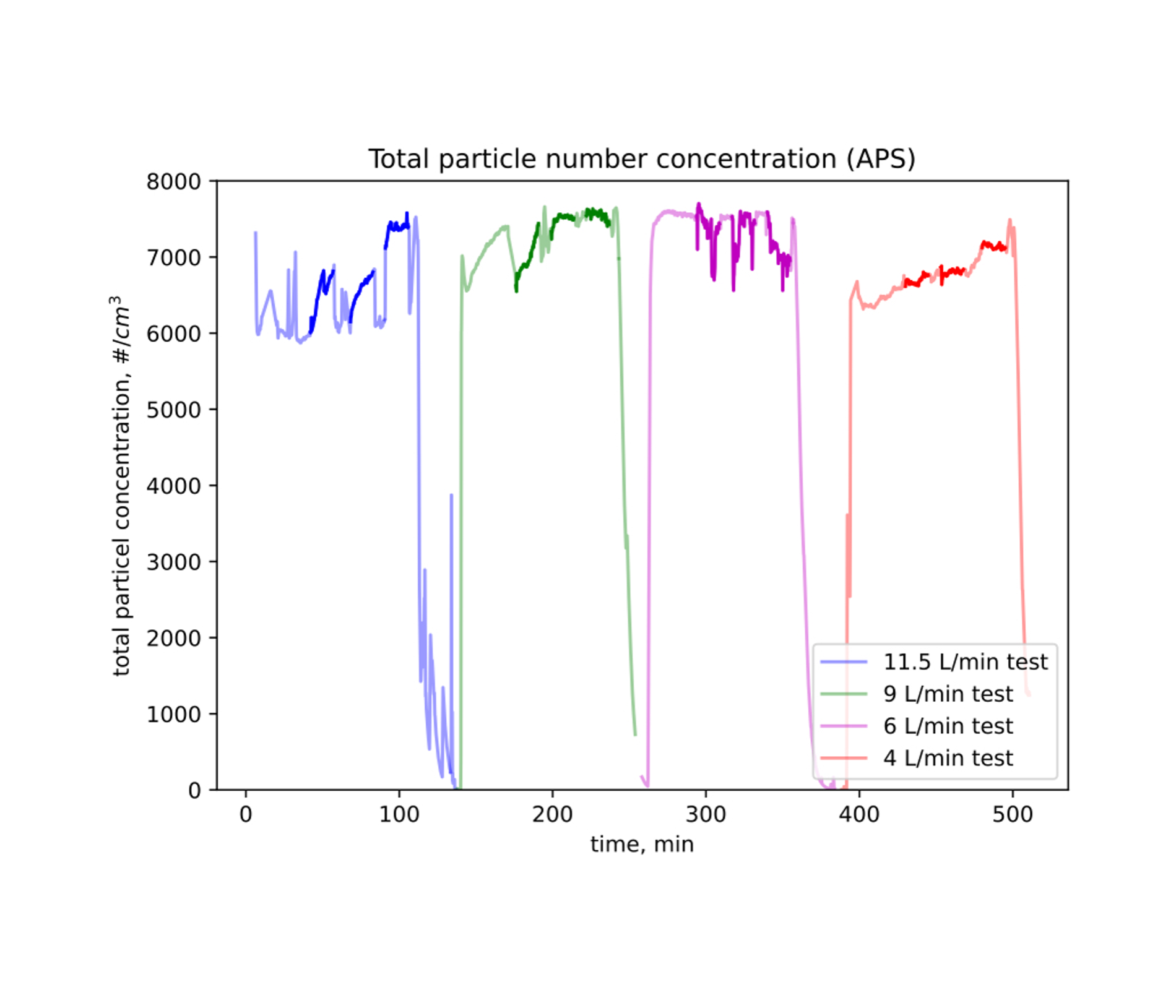

Supplement: S4 Fig — (TIFF) [file pone.0313604.s004.tiff]

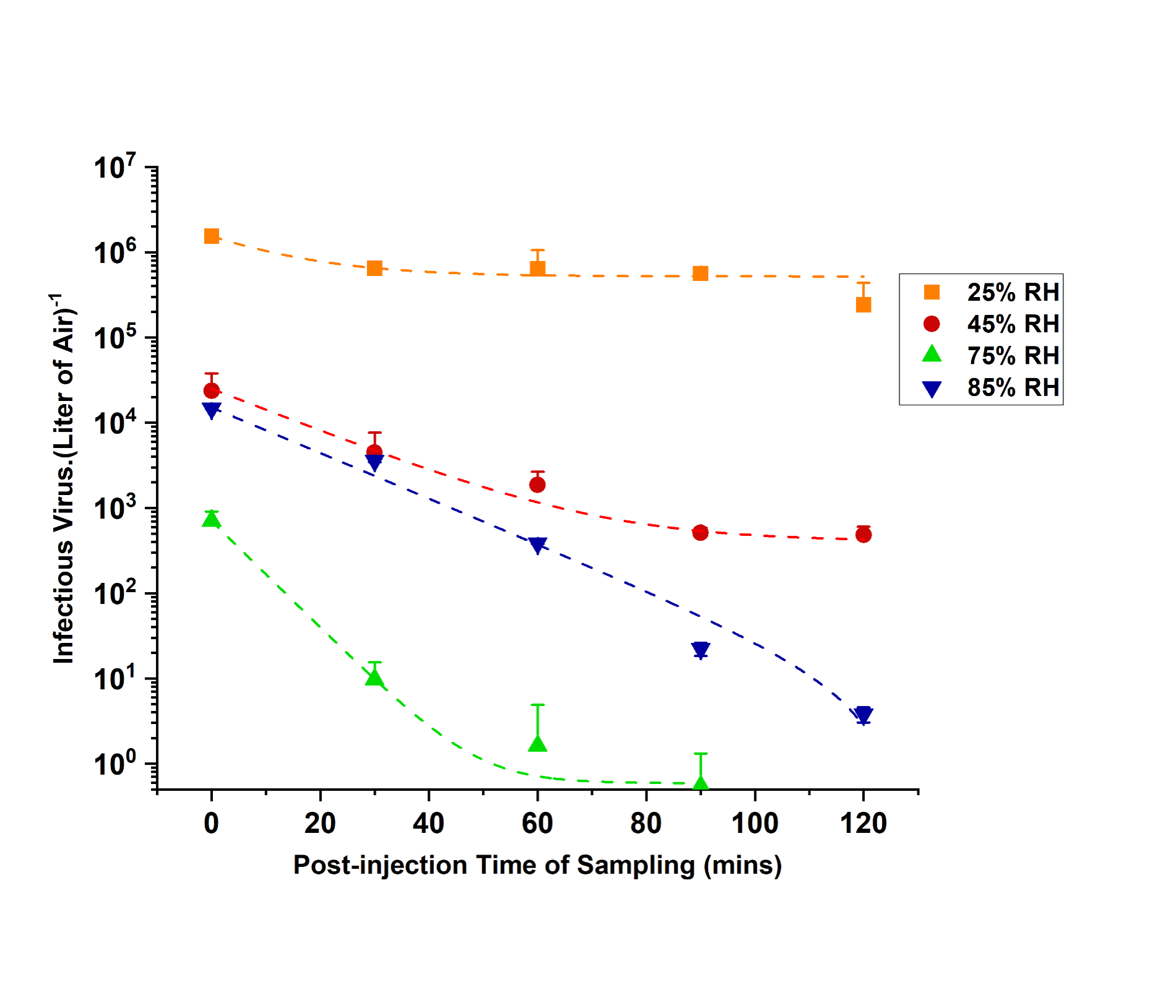

Supplement: S5 Fig — (TIFF) [file pone.0313604.s005.tiff]

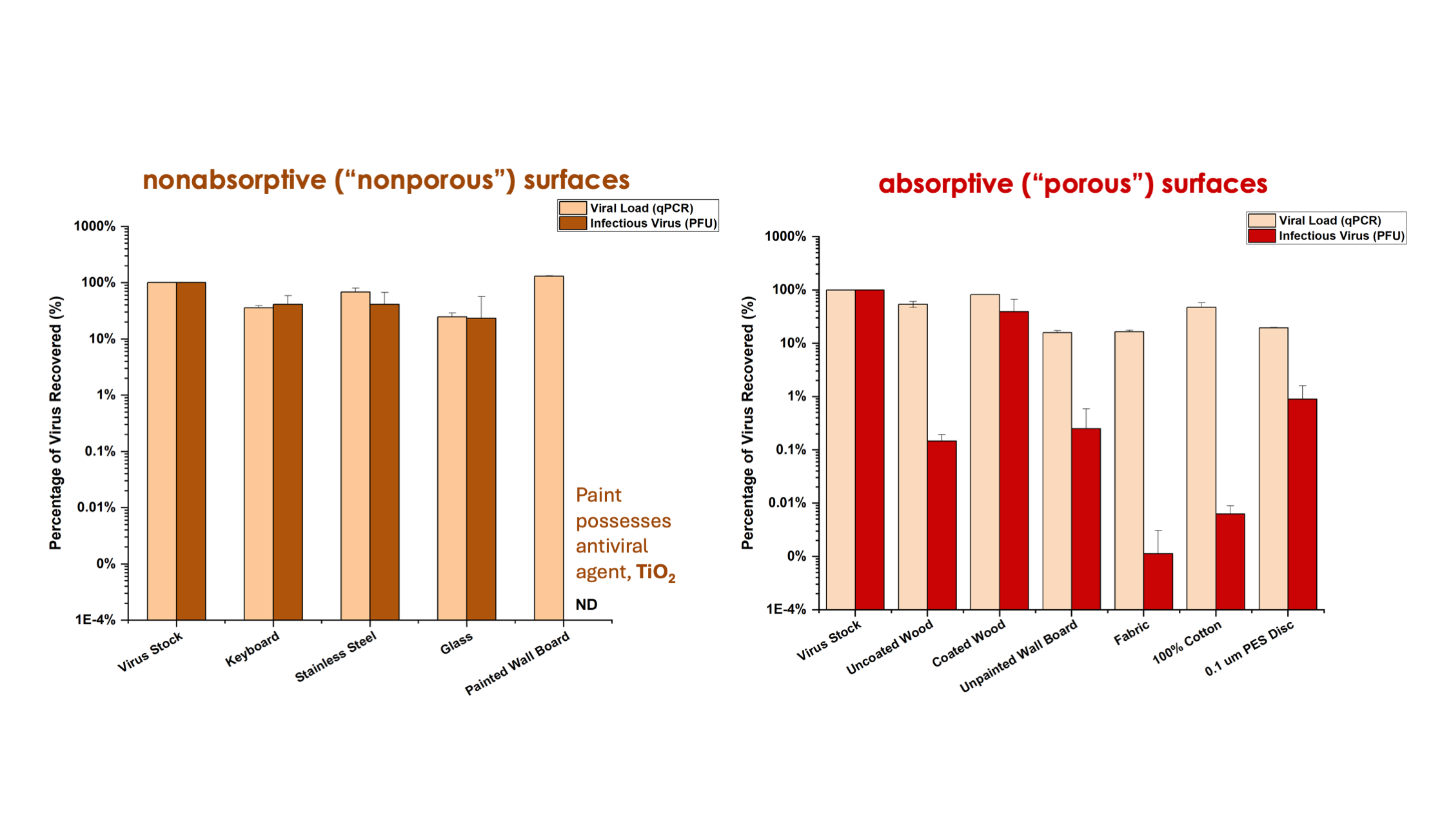

Supplement: S6 Fig — (TIFF) [file pone.0313604.s006.tiff]
